# The Cytocompatibility of Silver Diamine Fluoride on Mesenchymal Stromal Cells from Human Exfoliated Deciduous Teeth: An In Vitro Study

**DOI:** 10.3390/ma15062104

**Published:** 2022-03-12

**Authors:** David García-Bernal, Maria Pilar Pecci-Lloret, Sergio López-García

**Affiliations:** 1 Biochemistry, Molecular Biology and Immunology Department, Faculty of Medicine, University of Murcia, 30100 Murcia, Spain; david.garcia23@um.es; 2 Gerodontology and Special Care Dentistry Unit, Morales Meseguer Hospital, Faculty of Medicine, University of Murcia, 30100 Murcia, Spain; 3 Department of Stomatology, Faculty of Medicine and Dentistry, Universitat de Valencia, 46003 Valencia, Spain; sergio.lopez-garcia@uv.es

**Keywords:** silver diamine fluoride, cytotoxicity, SHEDs, cytocompatibility

## Abstract

Silver diamine fluoride (SDF) has been used for many years for the treatment of caries, and minimally invasive dentistry concepts have made it popular again. The fact that its application does not require the administration of anesthesia makes its use in children more desirable. The aim of this study was to determine the cytotoxicity of two new commercial SDF products: Riva Star (SDI Dental Limited) and e-SDF (Kids-e-Dental) on mesenchymal stromal cells from human exfoliated deciduous teeth (SHEDs). SHEDs were exposed to SDF products at different concentrations (0.1%, 0.01% and 0.005%). Then different assays were performed to evaluate their cytocompatibility on SHEDs: IC50, MTT, cell migration (wound healing), cell cytoskeleton staining, cell apoptosis, generation of intracellular reactive oxygen species (ROS), and ion chromatography. Statistical analyses were performed using one-way ANOVA and Tukey’s post hoc test (*p* < 0.05). Riva Star Step 2 showed the same cell metabolic activity when compared to the control condition at any time and concentration. Meanwhile, e-SDF displayed high cytotoxicity at any time and any concentration (*** *p* < 0.001), whereas Riva Star Step 1 displayed high cytotoxicity at any time at 0.1% and 0.01% (*** *p* < 0.001). Only e-SDF showed a statistically significant decreased cell migration rate (*** *p* < 0.001) at all times and in all concentrations. At 0.1%, e-SDF and Riva Star Step 1 only showed 4.37% and 4.47% of viable cells, respectively. These results suggest that Riva Star has better in vitro cytocompatibility on SHEDs than does e-SDF. Riva Star Step 1 was found to be as cytotoxic as e-SDF, but it had better biological properties when mixed with Riva Star Step 2. Our findings suggest that Riva Star is more suitable when used in deciduous teeth due to its lower cytotoxicity compared to e-SDF.

## 1. Introduction

Minimally invasive dentistry (MID) is an evidence-based concept that aims to preserve as much tooth structure as possible [1]. MID includes a variety of procedures such as resin infiltration, pit and fissure sealing, the Hall Technique, atraumatic restorative treatment (ART), and glass ionomer restorations. Along with preventive procedures such as frequent control of biofilms, dietary advice, and improvement of oral hygiene habits, as well as the application of fluoride varnishes and silver diamine fluoride (SDF) to decrease demineralization and improve remineralization of the area affected by caries [2,3], the use of MID procedures has increased in dentistry after the COVID-19 pandemic with the concept of “non-aerosol” [4,5,6].

Even though SDF has been used for many years for caries treatment in some countries [7], these new trends have made it popular again. The fact that its application does not require the administration of anesthesia if the clinician so decides (depending how much carious tissue is removed and which instruments are used) makes its use in children more simple [8]. In addition, the procedure is suitable for patients with special needs; for children whose age, dental anxiety, or behavioral or medical conditions do not allow a conventional treatment; in cases when parents are uncomfortable accepting general anesthesia or sedation for their children; and for children who have several cavities and cannot be treated in one session [8,9,10,11]. The application of SDF is painless and quick [9,12]. It is a minimally-invasive procedure for arresting untreated dental caries [12] and can help keep the tooth in position to maintain the mesio-distal space, since the tooth is the best space maintainer in primary or mixed dentition, avoiding extraction of the tooth [7]. SDF paralyzes caries, turning it into chronic caries without the need to remove affected dentine [13], and helps prevent the appearance of secondary caries with its use before restoration [14,15]. Reapplication of SDF annually to previously treated teeth is recommended to maintain its effectiveness. In patients with a high risk of caries development, it could even be applied every six months [16,17].

The protocol for its use is simple, following the manufacturer’s instructions: clean the tooth to reduce the amount of plaque, isolate the lips and skin with vaseline to prevent staining, and isolate the tooth. Then dry the surface and apply the SDF with a microbrush and let it dry [9,17]. It is essential to warn patients that SDF use leaves a black stain on the applied area [18,19] and may have other adverse effects, such as tooth and gum pain, gum swelling, or gum bleaching [17].

This material may be used to treat caries as long as the tooth is free of contraindications. These include pain, infection, radiographic signs of pulp involvement [17], silver allergy, and food packing, unless the shape of the tooth has been modified to avoid it and the patient is able to clean the tooth, or an ionomer cement or composite restoration is placed after its application. This is complementary to SDF application, since by itself it does not restore the tooth’s function or form [7,17,20].

It is essential to analyze the cytocompatibility of the dental materials used with patients [21,22]. This enables us to know the reaction of the mesenchymal stromal cells to these materials and determine if their toxicity is higher or lower and if it varies with time or concentration [23,24]. In the case of SDF, it will be placed relatively close to the dental pulp, which can affect the function and visibility of the mesenchymal stromal cells of deciduous teeth (SHEDs) [25]. To our knowledge there are only two studies about the cytotoxicity of SDF [26,27]. Thus, the aim of our study was to determine the cytocompatibility of two commercial SDF products on mesenchymal stromal cells from human exfoliated deciduous teeth (SHEDs). The null hypothesis was that there were no significant differences in terms of cytocompatibility between the different SDF products compared to untreated control cells.

## 2. Materials and Method

### 2.1. Preparation of SDF Eluates

The tested materials were two different commercially available SDF products: Riva Star and e-SDF. The manufacturer’s data, the composition, and the lot number of each of the materials tested are shown in Table 1. Due to the format of Riva Star, which has two separate solutions, it was decided to analyze them by separate or mixed at a 1:2 ratio, according to the manufacturer’s instructions. Eluates of these materials were prepared following ISO 10993-5 recommendations [23]. To prepare a 10% concentration, 1 mL of each SDF was blended with 9 mL of DMEM culture medium (Gibco, Thermo Fisher Scientific, Carlsbad, CA, USA) and filtered through a 0.22-µm syringe filter. Then, eluates were diluted with DMEM culture medium to generate different dilutions (0.1%, 0.01%, and 0.005%).

### 2.2. Isolation and Culture of Mesenchymal Stromal Cells from Deciduous Teeth

Human exfoliated deciduous teeth (healthy and free of caries) were collected from healthy children between six and nine years of age (*n* = 8). Previously, parents of the donor children signed an informed consent in accordance with the Helsinki Declaration Guidelines and the Institutional Review Board of the University of Murcia (reference no. 2199/2018). As reported previously [28], teeth were fragmented with a pincer to uncover the pulp tissue, which was minced in a sterile glass petri dish and digested with 3 mg/mL collagenase A (Sigma-Aldrich, St. Louis, MO, USA) at 37 °C for 1 h. Cells obtained after enzymatic digestion were seeded at 1.5 × 10^5^ cells/cm^2^ in culture flasks (Falcon, Corning, New York, NY, USA) in DMEM medium (Thermo Fisher Scientific) supplemented with 10% fetal bovine serum (Sigma-Aldrich, St. Louis, MO, USA), 1% GlutaMAXTM (Thermo Fisher Scientific), and 1% P/S (complete growth medium), and cultured at 5% CO_2_ and 37 °C for three days. The adherent cells were grown up to 80% confluence and were defined as passage zero (P0). For subsequent passaging, cells were washed with phosphate-buffered saline (PBS) (Gibco Invitrogen, Carlsbad, CA, USA) and detached with 0.25% trypsin-EDTA solution (Gibco Invitrogen) at 37 °C for 2–5 min. Culture complete medium was added to inactivate the trypsin activity. Finally, SHEDs were centrifuged for 5 min at 1200 rpm and seeded at a density of 5 × 10^3^ cells/cm^2^.

Before the cytotoxicity analysis of SDF, SHEDs were characterized by flow cytometry using specific antibodies for CD73, CD90, CD105, CD14, CD20, CD34, and CD45 (human MSC phenotyping cocktail; Miltenyi Biotec, Bergisch Gladbach, Germany) in a FACS Canto II flow cytometer (Becton Dickinson, Franklin Lakes, NJ, USA).

### 2.3. IC50 and MTT Assays

As reported previously [23], for cytotoxicity evaluation, the dose of each tested material that could decrease cell viability by 50% (half maximal inhibitory concentration (IC50)) was analyzed graphically by plotting the metabolic activity percentage on the Y-axis and the concentration percentage of each SDF product on the X-axis. In addition, IC50 values were analyzed by non-linear regression using GraphPad Prism software version 8.1.0 (GraphPad Software Inc, San Diego, CA, USA).

Also, 1 × 10^4^ SHEDs were cultured with different dilutions of the material eluates to analyze cell metabolic activity after 24, 48, and 72 h of culture by 3-[4,5-dimethylthiazol-2-yl]-2,5 diphenyl tetrazolium bromide (MTT) assay. Briefly, the MTT reagent (Sigma-Aldrich) was added to the wells and incubated for 4h. Then dimethylsulfoxide (DMSO) (Sigma-Aldrich) (100 μL/well) was added to solubilize the formazan dye. Finally, absorbance at 570 nm wavelength was measured in a microplate reader (ELx800; Bio-Tek Instruments, Winooski, VT, USA). Each experimental condition was performed in quintuplicate and replicated in three separate experiments.

### 2.4. Cell Migration

To evaluate the biological effects of the different SDF eluates on cell migration, wound healing assays were carried out as previously described [29]. SHEDs were seeded at a density of 2 × 10^5^ cells/well and cultured for 24 h at 37 °C to obtain confluent cell monolayers. Next, using a sterile pipette tip, a wound or scratch was performed in each cell monolayer, washed twice with PBS to discard unattached cells after scratching, and cultured in complete growth medium alone (control) or in complete growth medium containing the different SDF eluates at 0.1%, 0.01%, and 0.005% concentrations at 37 °C for 24, 48, or 72 h. Finally, ImageJ software (National Institutes of Health, Bethesda, MD, USA) was used to measure the percentage of open wound area at each time point relative to the same wound area at 0 h in the same well. Three independent experiments were done, each performed in triplicate for each SDF eluate and concentration.

### 2.5. Cell Cytoskeleton Staining

To examine possible changes in cell morphology and in F-actin cytoskeleton content and organization, phalloidin staining was carried out, as previously described [29]. Briefly, 3 × 10^4^ SHEDs were cultivated on glass coverslips, allowed to stick and spread, and cultured in complete growth medium alone (control) or in complete growth medium containing the different SDF eluates at 0.1%, 0.01%, and 0.005% concentrations for 72 h at 37 °C. Then the glass coverslips were washed twice with PBS at 37 °C, fixed in 4% formaldehyde in PBS for 10 min, permeabilized in 0.25% Triton X-100 solution (Sigma-Aldrich) for 5 min, and washed 3 times with PBS. F-actin cytoskeleton and nuclei were then stained with AlexaFluor™594-labeled phalloidin (ThermoFisher Scientific, Carslbad, CA, USA) and 4,6-diamidino-2-phenylindole dihydrochloride (DAPI) (ThermoFisher Scientific), respectively, at r/t in the dark for 30 min. Finally, immunofluorescence images were acquired in a Leica TCS SP2 confocal microscope (Leica, Wetzlar, Germany). Each of the SDF eluate concentrations was analyzed in three independent experiments in triplicate.

### 2.6. Apoptosis/Necrosis and ROS Assay

SHED viability and intracellular reactive oxygen species (ROS) production were analyzed after exposure to the different SDF eluates by annexin-V/7-aminoactinomycin D (7-AAD) and the general oxidative stress indicator CM-H2DCFDA staining, respectively, as previously described [23,29]. Briefly, SHEDs were cultured in complete culture medium alone (control), or in complete medium containing the different SDF eluates at 0.1%, 0.01%, and 0.005% concentrations for 72 h at 37 °C. Afterwards, the cells were washed and stained with FITC-labeled annexin-V and 7-AAD (Immunostep, Salamanca, Spain) for 15 min at r/t following the manufacturer′s recommendations, or with 5 µM CM-H2DCFDA (Molecular Probes, Eugene, OR, USA) for 30 min at 37 °C. Finally, SHEDs were acquired in a BD FACS CantoTM flow cytometer (BD Biosciences, San Jose, CA, USA), and percentages of live and apoptotic/necrotic cells or CM-H2DCFDA positive cells were analyzed with FlowJo software (FlowJo LLC, Ashland, OR, USA). All experimental conditions were replicated independently in triplicate and analyzed in three separate experiments.

### 2.7. Ion Chromatography

Anion concentrations of fluoride in the different SDF eluates were measured using a Dionex ICS-2100 ion chromatography (Thermofisher, Sunnyvale, CA, USA) (IC) with an AS19 column and potassium hydroxide as eluent, as previosly described [30]. Instrument settings and data acquisition were controlled using Chromeleon^®^ software v7.3.1. The ICS-2100 instrument was used in conjunction with a Dionex AS autosampler (Thermofisher, Sunnyvale, CA, USA). A 50 µL sample loop was employed for all the analyses. The lCS-2100 system was used for the separation and suppressed conductivity detection of anions. Separation was performed on a 4-mm Dionex IonPac AS19 column (Thermofisher, Sunnyvale, CA, USA) (250-mm, 4-mm ID) using a Dionex AG19 guard column (Thermofisher, Sunnyvale, CA, USA) (50-mm, 4-mm ID), coupled with a Dionex ASRS300, 4-mm suppressor (Thermofisher, Sunnyvale, CA, USA). Hydroxide eluent gradients were generated online using the Dionex EluGen III KOH cartridge (Thermofisher, Sunnyvale, CA, USA). A continuously regenerated anion trap column (Dionex CR-ATC (Thermofisher, Sunnyvale, CA, USA)) was plumbed inline after the eluent generator cartridge. The optimized hydroxide eluent gradient was: 0–10 min: 10 mM isocratic; 10–25 min: gradient from 10 to 45 mM. Standard solutions of inorganic anion were purchased as different ppm anionic concentration standards from Sigma-Aldrich and were diluted as required with Milli-Q distilled water. Water treated with a Millipore (Bedford, MA, USA) Milli-Q system was used to prepare standard solutions and eluents.

### 2.8. Statistical Analysis

The experimental results are presented as the mean ± standard deviation (SD). Data were statistically analyzed by one-way analysis of variance (ANOVA) followed by Tukey’s post-hoc test for multiple comparisons using GraphPad Prism software v8.1.0 (Graph-Pad Software v6.01). *p* < 0.05 was considered to indicate a statistically significant difference.

## 3. Results

### 3.1. SHEDs Phenotypic Characterization

**Figure 1:**
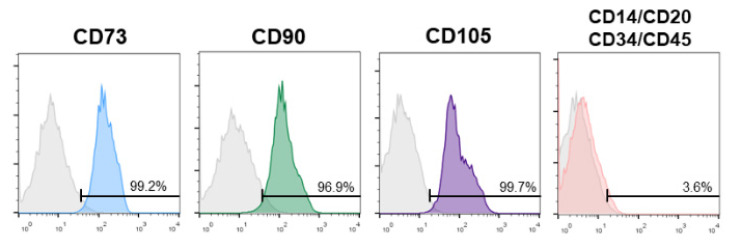
SHED mesenchymal stem cell phenotype analysis by flow cytometry with specific antibodies for the mesenchymal surface markers CD73, CD90, and CD105, and the hematopoietic markers CD14, CD20, CD34, and CD45. Control isotype staining is also shown (grey histograms). Numbers inside histograms represent the percent of positive cells for each marker.

In all the SHED samples tested, the mesenchymal stem cell surface molecules CD73 (99.2%), CD90 (96.9%), and CD105 (99.7%) were expressed, while the expression of the hematopoietic markers CD34 and CD45 was lower than 5% (Figure 1).

### 3.2. IC50 and MTT

**Figure 2:**
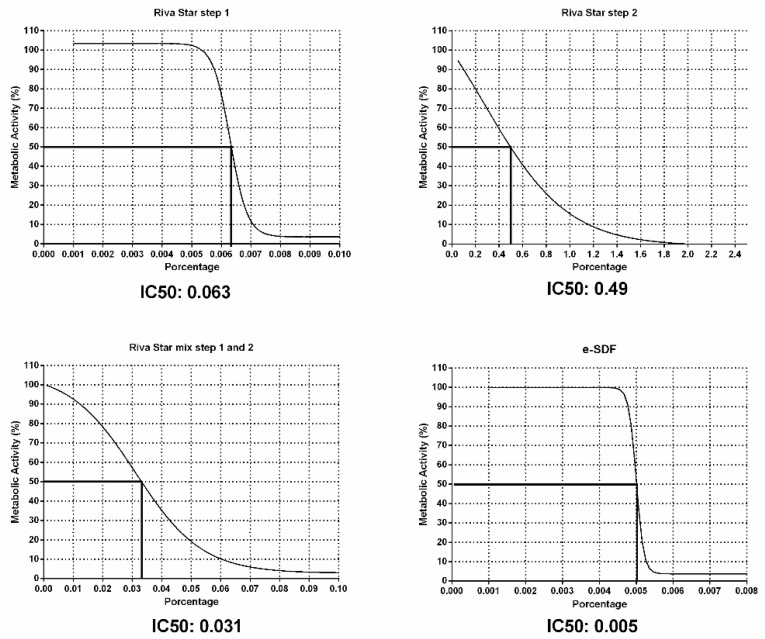
Calculation of IC50 values of the various SDF. The percent of concentration of each SDF in the extract was calculated for 50% inhibition of SHED metabolic activity. Data were analyzed by non-linear regression by plotting the percentage of the metabolic activity on the *y*-axis and the percentage of each varnish on the *x*-axis. Curves shown are representative from *n* = 3 separate experiments.

IC50 values (percentage concentration of each SDF product to inhibit 50% of SHEDs viability) were the following: Riva Star Step 1: 0.063%; Riva Star Step 2: 0.49%; Riva Star mix Step 1 and Step 2: 0.031%; and e-SDF: 0.005% (Figure 2).

**Figure 3:**
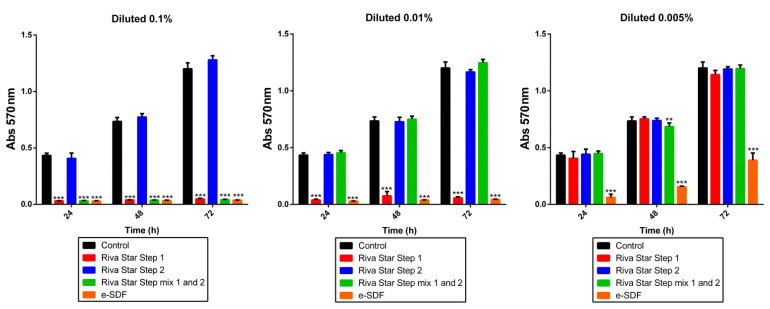
MTT assay. Metabolic activity of SHEDs after exposure to different concentrations of SDF products for 24, 48, and 72 h. Absorbance values at 570 nm were significantly different from the control group (** *p* < 0.01; *** *p* < 0.001) according to one-way ANOVA and Tukey’s post hoc test.

Next, the cell metabolic activity was measured by MTT assay (Figure 3). At 0.1% concentration, only Riva Star Step 2 showed the same cell metabolic activity when compared to the control condition at any time. Nevertheless, Riva star Step 1, Riva Star mix Step 1 and Step 2, and e-SDF displayed a significant reduction of SHED metabolic activity at any time (*** *p* < 0.001). However, at 0.01% concentration, Riva Star Step 2 and Riva Star Mix Step 1 and Step 2 displayed the same metabolic activity as the control at any time, and while using Riva Star Step 1 and e-SDF a statistically significant metabolic activity reduction compared to the control was found (*** *p* < 0.001). Finally, using the different eluates at 0.005% concentration, only e-SDF showed a high cytotoxicity effect when compared to the control at any time (*** *p* < 0.001).

### 3.3. Cell Migration

**Figure 4:**
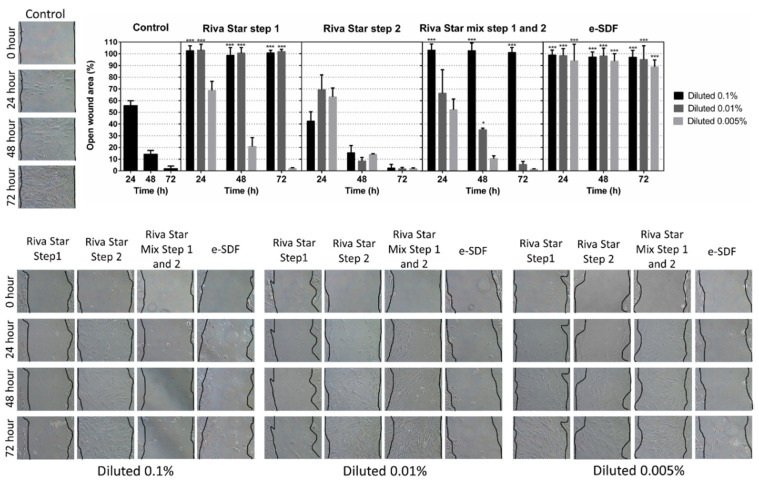
Migration of SHEDs after treatment with different SDF concentrations was analyzed by wound healing assays. Confluent SHED monolayers were cultured with complete growth medium (control) or different concentrations (0.1%, 0.01% and 0.005%) of the indicated SDF products for 72 h. Cell migration was expressed as the percentage of open wound area at each time point relative to the same wound area at 0 h (100%). Migration was significantly reduced compared to control (*** *p* < 0.001) according to one-way ANOVA and Tukey’s post hoc test.

Open wound areas of migrating SHEDs in the presence of different concentrations of SDF were measured after 24, 48, and 72 h of culture (Figure 4). Only Riva Star Step 2 showed comparable results on cell migration to those observed in the control conditions at any time and dilution. Conversely, only e-SDF showed statistically significant decreased cell migration rates (*** *p* < 0.001) at all times and all concentrations. Riva Star Mix Step 1 and Step 2 only showed significant differences at 0.1% at any time (*** *p* < 0.001) and at 0.01% at 48 h (* *p* < 0.05), while Riva Star Step 1 only showed a migration comparable to the control at 0.005% at any time. These findings support the findings observed in the MTT assays.

### 3.4. Cell Cytoskeleton Staining

**Figure 5:**
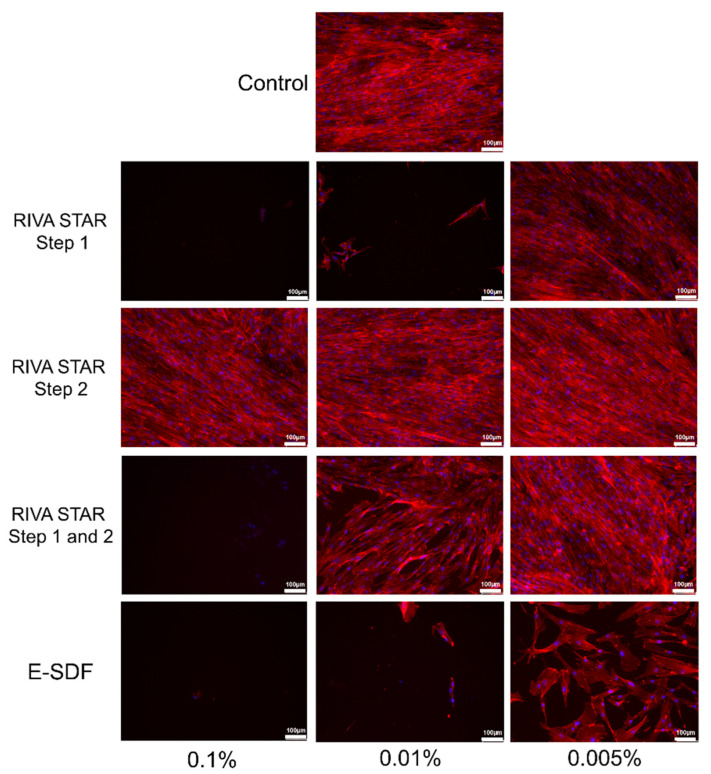
Morphological aspects and cytoskeleton F-actin fibers organization on SHEDs cultures exposed to the indicated SDF by confocal fluorescence microscopy. F-actin fibers were stained with AlexaFluor™594-labeled phalloidin (Thermo Fisher, Carslbad, CA, USA) (red fluorescence); meanwhile, cell nuclei were counterstained with DAPI (blue fluorescence). Images shown are representative from three independent experiments carried on in triplicate for each material. Scale bar: 100 μm.

After 72 h of exposure of SHEDs to SDF extracts (Figure 5), Riva Star Step 2 allowed the proliferation of a high number and well-attached cells at all concentrations, with SHEDs showing a fibroblastic morphology and high F-actin fibers, similar to what was observed in the control cells. However, Riva Star Mix Step 1 and Step 2 displayed some scattered cells at 0.01% and similar amounts and similar cell morphology to those observed in the presence of Riva Star Step 2 at 0.005%. Finally, e-SDF and Riva Star Step 1 did not show attached cells at 0.1% and 0.01%, but at 0.005% showed a lower number of attached SHEDs with an aberrant morphology.

### 3.5. Apoptosis/Necrosis Assay and ROS Assay

**Figure 6:**
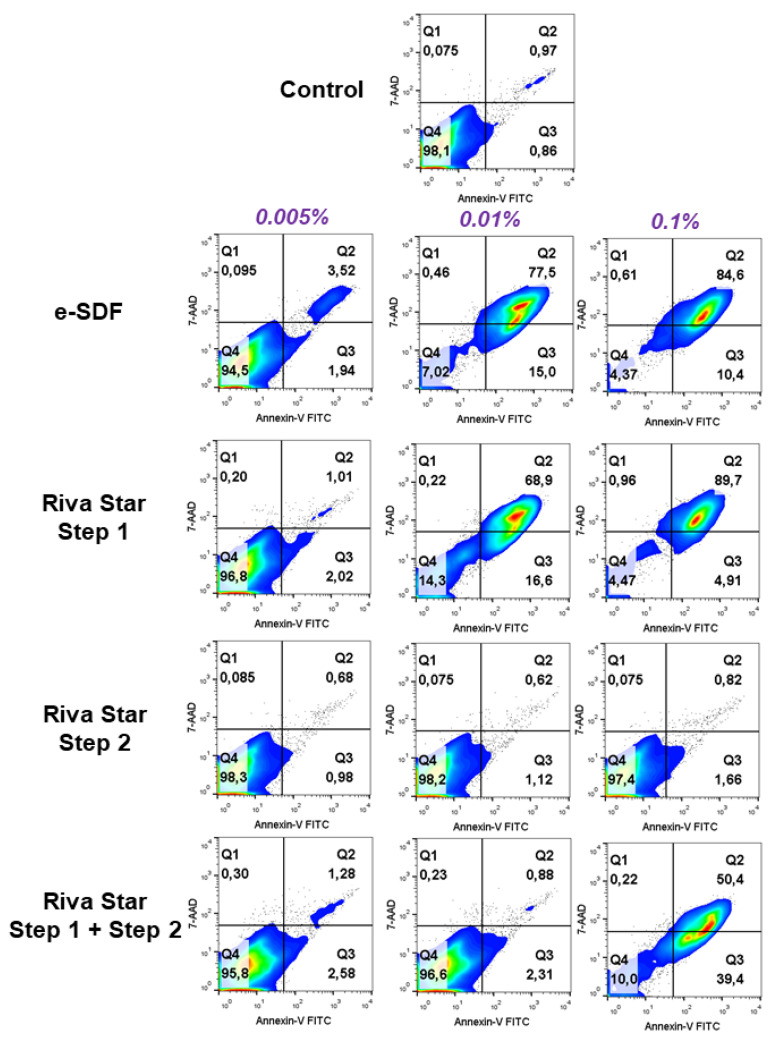
Flow cytometry analysis of cell apoptosis and necrosis induced by the different SDF concentrations on SHEDs by Annexin-V-FITC and 7-AAD staining. Numbers inside density plots represent percentages of live (Q4), early apoptotic (Q3), and late apoptotic necrotic cells (Q1 and Q2) at different concentrations (0.1%, 0.01% and 0.005%) and are representative from three independent experiments carried on in triplicate for each material.

As shown in Figure 6, at 0.005% the percentage of viable cells was: e-SDF 94.5%, Riva Star Step 1 96.8%, Riva Star Step 2 98.3%, and Riva Star Mix 95.8%. At 0.01%, only Riva Star Step 2 and Riva Star Mix Step 1 and Step 2 displayed similar levels of viable cells compared to control (98.2% and 96.6%, respectively), while e-SDF and Riva Star Step 1 only showed 7.02% and 14.3% of viable cells. At 0.1% e-SDF and Riva Star Step only showed 4.37% and 4.47% of viable cells, with >85% and >90% of late apoptotic and necrotic cells. Finally, Riva Star Mix Step 1 and Step 2 at 0.1% showed a decreased percentage of viable cells to 10% and an increased percentage of late apoptotic and necrotic cells up to 51%.

**Figure 7:**
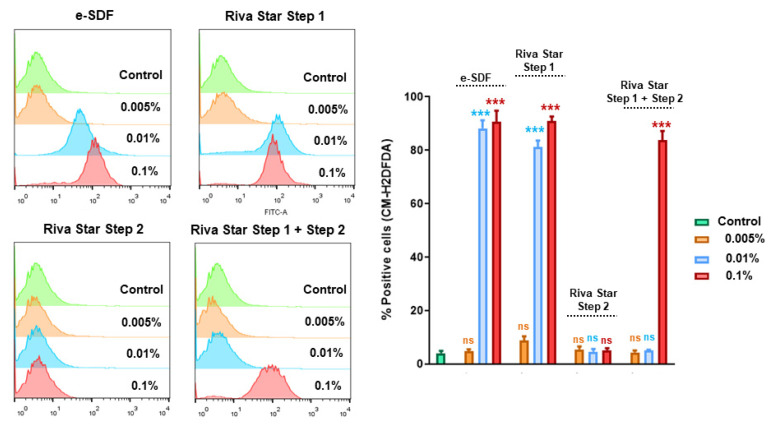
Analysis of intracellular reactive oxygen production (ROS) after treatment with different dilutions of the indicated SDF products by CM-H2DCFDA staining. Representative histograms obtained in each experimental condition are shown. Bar graphs show the quantification of positive CM-H2DCFDA SHEDs obtained with each resin extract dilution and are represented as mean ± SD from three independent experiments performed in triplicate. Percentages of CM-H2DCFDA-positive cells were significantly increased compared to the control,*** *p* < 0.001; *ns:* no significant; respectively, according to one-way ANOVA and Tukey’s post hoc test.

Intracellular ROS levels were measured in SHEDs cultures in the presence of e-SDF, Riva Star Step 1, Riva Star Step 2, and Riva Star Mix Step 1 and Step 2 at different concentrations (0.1%, 0.01%, and 0.005%) (Figure 7). When SHEDs were cultured with Riva Star Step 2 (0.1%, 0.01%, and 0.005%), there were no differences at any concentration compared to ROS levels observed in control cells. However, SHEDs cultured in the presence of e-SDF and Riva Star Step 1 eluates at 0.1% and 0.01% concentrations showed significantly increased ROS levels compared to control cells (*** *p* < 0.001). Finally, ROS levels in SHEDs exposed to Riva Star Mix Step 1 and Step 2 at 0.1% concentration were augmented compared to the control cells (*** *p* < 0.001).

### 3.6. Ion Chromatography

Fluoride concentration found in the different SDF products is shown in Table 2. Without any dilution, e-SDF has 7.38 gr/L of fluoride, Riva Star Step 1 has 7.04 gr/L, Riva Star Step 2 has <0.3 ppm, and Riva Star Mix Step 1 and Step 2 has 2 gr/L, so that e-SDF and Riva Star Step 1 contain a similar fluoride concentration. Riva Star Step 2 had <0.3 ppm of fluoride in all concentrations. At 0.1% e-SDF showed 73.77 mg/L, followed by Riva Star Step 1 with 70.35 mg/L, and Riva Star Mix Step 1 and Step 2 with 20.04 mg/L. At 0.01% e-SDF showed 6.99 mg/L, Riva Star Step 1 6.52 mg/L, and Riva Star Mix Step 1 and Step 2 1.93 mg/L. Finally, at 0.005% e-SDF showed 3.46 mg/L, Riva Star Step 1 showed 3.15 mg/L, and Riva Star Mix Step 1 and Step 2 showed 0.77 mg/L.

## 4. Discussion

This study aimed to analyze the in vitro biological properties of two commercially available SDF products: e-SDF and Riva Star. Different results have been found in both SDF products, which may potentially be influenced by the form of administration of the products, since e-SDF is applied in one step and Riva Star in two steps. Riva Star consists of two solutions (Step 1 and Step 2). All the results obtained in the present investigation showed that Step 1 displayed less cytocompatibility than Step 2, being comparable to the results obtained in e-SDF; meanwhile, when the mix of Step 1 and Step 2 is observed, the cytocompatibility results improve in all experiments. This could be explained by the composition of Riva Star Step 2 (see Table 1). This is a saturated potassium iodide (KI) solution. The solution is biocompatible, but when it is added to Riva Star Step 1 the MTT results were improved, showing a better cytocompatibility. This could suggest that KI in SDF could has a protective effect on cells, but this effect has not been previously described and more studies are needed to corroborate it.

Currently, only two studies analyze the cytocompatibility of SDF: Kim et al. [27] tested the cytotoxicity of these products using rat pulpal cells, and Fancher et al. [26] used human gingival fibroblasts. An essential aspect when selecting resources to be used during cytotoxicity testing is the cells and tissues likely to be affected by the tested agent. Previous studies showed since that human cell lines are more sensitive to these tests than animal cell lines, such as L929 [31,32], SDF should not be in contact with human gingival fibroblasts. However, there is a potential risk of accidental soft tissue contact, so it is important to possess this information. In addition, SDF is placed in the dentin surface affected by caries, near the pulp [9,16]; this is the reason that we used SHEDs.

Kim et al. [27] concluded that all tested SDF dilutions had a remarkable cytotoxic effect, but when they added reduced glutathione, it had a protective action, and the cytotoxicity decreased. This means that depending on the composition of the SDF, the cytotoxicity could change, as our results showed that when Riva Star Step 1 is combined with Riva Star Step 2 (a saturated solution of potassium iodide), the cytotoxicity decreased. More studies are needed to corroborate this.

Fancher et al. [26] analyzed the cytotoxicity of other SDF such as Advantage Arrest. They concluded that this product is cytotoxic to gingival fibroblasts in a concentration of 0.01%. In our study, and in agreement with these results, e-SDF was cytotoxic towards SHEDs at 0.01% concentration at any time; even so, it was cytotoxic in lower concentrations, such as 0.005%. Conversely, Riva Star Mix Step 1 and Step 2 were only cytotoxic at 0.1% concentration, obtaining the same results as those in the control group at 0.01%.

The different tests were carried out at different concentrations to resemble clinical situations in which the carious lesion is closer or further away from the pulp. Clinically, this means that it is very important to take into account the distance to the pulp. When SDF products are closer to the pulp, a situation that we reproduce with the highest concentrations in our experiments, it would be more cytotoxic for SHEDs. Even so, in the wound healing assays, SHED migration rates were different between the products, times, and concentrations, and we observed that at higher concentrations the SDF products delay the healing process. In addition, the apoptosis/necrosis assay showed that a higher concentration increased the late apoptotic necrotic cells, and fewer live cells decreased the healing process of the wound healing assay, being in concordance with it. This could have negative consequences if there is an inflammatory process of pulp tissue, such as reversible pulpitis, and SDF reaches the pulp and hinders its healing. As our results showed, this means that Riva Star use (Riva Star 1 and Riva Star 2) will be preferable compared to e-SDF use due to its better cytocompatibility.

Fluoride concentrations were also measured. Neither Riva Star nor e-SDF had the expected concentration stated by the manufacturer. Patel et al. [33] analyzed the fluoride concentration of seven types of SDF products. None of the SDF fluoride concentrations matched with the expected fluoride concentration indicated by the manufacturer. Some of them had a higher fluoride concentration and others had a lower one. Soares-Yoshikawa et al. [34] also analyzed the fluoride concentration in six different SDF products, and their results also did not match the manufacturer’s specifications. These two articles were in agreement with our results, which suggests a need for precise controls in the manufacturing process of this type of product.

## 5. Conclusions

The results from the present study suggest that Riva Star has better in vitro cytocompatibility than e-SDF. Riva Star Step 1 was as cytotoxic as e-SDF, but its biological properties were improved when mixed with Riva Star Step 2. This suggests that the use of Riva Star (Step 1 + Step 2) is preferable when used in deciduous teeth due to its lower cytotoxicity compared to e-SDF.

## Figures and Tables

**Table 1:** Material tested.

Material	Manufacturer	Composition	Lot Number
**Riva Star**	SDI, 3-15 Brunsdon Street, Victoria 3153, Bayswater, Australia	Step 1 (Silver capsule): 30–35% silver fluoride and >60% ammonia solution. Step 2 (Green capsule): saturated potassium iodide solution	11508613
**e-SDF**	Kids-e-Dental, Akruti Arcade, Headquarters 411, JP Rd, opp. A.H. Wadia school, Azad Nagar, Andheri West, Mumbai, Maharashtra, 400053, India	Silver diamine fluoride contains approximately 24–28% (weight/volume) silver and 5–6% (weight/volume), ammonia solution	ESDF JK 122

**Table 2:** Product fluoride concentration.

Product	Fluoride Concentration
e-SDF 0.005%	3.4657 mg/L
e-SDF 0.01%	6.9901 mg/L
e-SDF 0.1%	73.7795 mg/L
Riva Star Step 1 0.005%	3.185 mg/L
Riva Star Step 1 0.01%	6.5263 mg/L
Riva Star Step 1 0.1%	70.3579 mg/L
Riva Star Step 2 0.005%	<0.3 ppm
Riva Star Step 2 0.01%	<0.3 ppm
Riva Star Step 2 0.1%	<0.3 ppm
Riva Star Mix Step 1 and Step 2 0.005%	0.779 mg/L
Riva Star Mix Step 1 and Step 2 0.01%	1.9342 mg/L
Riva Star Mix Step 1 and Step 2 0.1%	20.0438 mg/L
e-SDF	7.38 gr/L
Riva Star Step 1	7.04 gr/L
Riva Star Step 2	<0.3 ppm
Riva Star Mix Step 1 and Step 2	2 gr/L

## Data Availability

The data presented in this study are available on request from the corresponding author.
